# Immunohistochemical Expression of MMP-9 and E-Cadherin in subtypes of Ameloblastoma

**DOI:** 10.12669/pjms.38.1.4465

**Published:** 2022

**Authors:** Farah Farhan, Zainab Niazi, Sana Masood, Beenish Abbas

**Affiliations:** 1 Dr. Farah Farhan, M.Phil (Oral Pathology), Assistant Professor Foundation University College of Dentistry, Islamabad, Pakistan; 2 Dr. Zainab Niazi, M.Phil (Oral Pathology), Assistant Professor, Islamabad Medical and Dental College, Islamabad, Pakistan; 3 Dr. Sana Masood, MSc, DPH, BDS (Community Dentistry), Assistant Professor Foundation University College of Dentistry, Islamabad Pakistan; 4 Dr. Beenish Abbas, FCPS (Operative Dentistry), Assistant Professor Foundation University College of Dentistry, Islamabad Pakistan

**Keywords:** Ameloblastoma, E-Cadherin, Matrix metalloproteinases, Histopathology

## Abstract

**Background & Objective:**

Ameloblastomas have been analyzed histologically for diagnostics and its sub-classification; however, the analysis carried out so far does not provide any authentic evidence regarding prognosis of Ameloblastoma. Subject categorization is still a topic of debate. The purpose of this study was to determine the immuno-expression of markers such as MMP-9 and E-Cadherin in different sub-types of ameloblastoma and establish their correlation with histological variants.

**Methods:**

Analytical study of 71 cases of ameloblastoma was conducted in AFIP Rawalpindi, between January to June, 2019. Samples were taken from different intraoral sites including the patients with tumor of ameloblast. The tumor was sub classified histologically on the basis of WHO classification. ‘Chi Square’ Test was applied to find the association of MMP-9 and E-Cadherin with histological variants of ameloblastoma. P-value ≤ 0.05 were found statistically significant.

**Results:**

On histopathological sub-classification, 52.1% were diagnosed as follicular type, 23.9% were plexiform type, 14.1% were Acanthomatous type and 9.9% were of unicystic ameloblastoma. 80% of the total Acanthomatous type and 59% of the total plexiform had strong immuno-expression, which was significantly different from follicular type MMP-9 (p ≤ 0.05). All cases of unicystic ameloblastoma and 67.6% of follicular type showed strong immuno-expression significantly different from 20% of Acanthomatous type and 59% of plexiform type E-Cadherin (p ≥ 0.05). On the other hand, when statistical analysis was carried out, an inverse relation between MMP-9 and E-cadherin was observed.

**Conclusion:**

The effect of MMP-9 and E-cadherin in ameloblastoma is aggressive in nature and effectiveness was seen in subtypes of ameloblastoma.

## INTRODUCTION

Ameloblastoma is known as benign, slowly growing and locally invasive tumor associated with highly destructive recurrence risk and may require radical surgery. Contributing for less than 1% of all tumors of the jaw, ameloblastoma is a relatively rare dental tumor.^1^ Amongst white and black male/female, the yearly incidences reported per million are 0.44, 0.18, 1.96 and 1.20 respectively.^2^ In another study conducted at Karachi, Pakistan regarding the prevalence of odontogenic tumors and cysts, ameloblastoma was the second most common tumor reported out of 141 cases of odontogenic tumors over a period of 10 years.^3^

The World Health Organization (WHO) clinical categorization of tumor of head and neck classified benign ameloblastoma into three different types: unicystic, extraosseous and peripheral. Histopathologically, it can be further subdivided into follicular, desmoplastic, basal cell, plexiform, acanthomatous, and granular cells.^4^

Matrix Metalloproteinases (MMPs) fit into zinc reliant endopeptidases. The MMPs are formed as inactive precursors holding a propeptide and a secretory signal sequence. Proteolytic cleavage of this propeptide is essential for MMP activation. Carrying the label of enzyme, MMP-9 is encoded by MMP-9 gene in human.^5^ It breaks down the type IV collagen which is responsible for the spread of tumor.^6^

Cadherin originates from a family of glycosylated calcium dependent adhesion molecules. They are known as single-pass trans-membrane proteins. There are more than eighty known members of the cadherin super-family.^7^ E-cadherin enjoys as one of the most important members of this family and is known to be essential element because it has the capability of suppressor of invasion properties.^8^

This study was conducted to determine the expression of immuno-histochemical markers including E-Cadherin and MMP-9 in different histological types of ameloblastoma for gauging and predicting the aggressiveness and behavior of this tumor in our population. These markers play a significant role in local invasiveness.

## METHODS

Sequel to acquiring approval from Institutional Review Board (IRB) of AFIP, Rawalpindi Pakistan vide letter N0. 17/AFIP/IRB.2018, subject analytical study was carried out between January to June, 2019. With a written informed consent, a sample of 71 cases of ameloblastoma (calculated by WHO sample size calculator, n = Z^2^ PQ/ d^2^, where Z^2^ = 3.8416, P=5% (0.05), Q=1-P (0.95), d^2^ = 0.0025),^9^ Paraffin-embedded blocks of both previously and freshly diagnosed cases of ameloblastoma were taken from any intraoral site. Necrosed, scanty and autolysed tissues, poorly oriented tissue, previously treated cases having bone resorption and with skin involvement (recurrent cases) were excluded in the study.

Retrieval of the paraffin blocks along with their histopathology request forms were provided with the specimens at the time of submission. After confirmation of diagnosis, classification according to the World Health Organization (WHO)^4^ and histopathological sub-typing was done on freshly prepared slides. MMP-9 and E-cadherin were applied on the tissue according to standard protocol. The intensity of the stain was measured according to WHO classification. Immuno-reactivity was evaluated and its association with histopathological types was carried out.

### Histological technique:

In this method, biopsy was taken by applying 10% buffered formalin on all specimens in specially designed perforated walled plastic cassettes and placed in Tissue Tek VIP-5 imported processor. The sample was dehydrated with alcohol and then cleared with xylene, after which, impregnation of tissues with extreme temperature of 52-55^°^C was done. Process of impregnation required molten paraffin wax which was casted using tissue processor and then allowed to cool. Finally, the molten piece was sectioned into small sections using cut rotary machines, Rotary Microtome SRM 200-1 (Sakura, Japan). These slices are frosted and stained by an instrument called Varistain Multiproy Slide Stainer (Shandon, UK).

### Haematoxylin and Eosin Staining:

In this technique, drying phenomenon was done at 37°C for almost two to four hours at 58 degrees and fixed on glass slide and dipped in xylol for almost three minutes. It was then transferred into alcohol with addition of rectified spirit and methyl spirit which took around three minutes. Besides, it was also washed with water for two minutes. Afterwards, the slides were dipped into haematoxylin and washed in running water for thirty seconds. After repeated washings, slides were then placed into ammonia until litmus paper changed its color to blue. The slides were then dipped into Eosin Stain and dehydrated with alcohol and clearing with ethanol and finally it was mounted.^10^

### MMP-9 and E-Cadherin Application Techniques:

Polycolonal antibody to MMP-9 (Code A0150; Dako, Denmark) and Monocolonal antibody to E-Cadherin (Catalogue no: 081223; Invitrogen, USA) were used at a concentration of 1:100. Slides were dipped with peroxidase solution and rinsed with PBS followed by application of serum blocking solution. Principle antibody incubated for about 30 to 60 minutes at room temperature and was again rinsed with PBS. Finally, chromogen was applied, incubated for 5-10 minutes at room temperature and then rinsed with PBS.

### Immuno-Histochemical Staining and Scoring:

Immuno-reactivity of MMP-9 and E-Cadherin was evaluated on the criteria described by Alves Pereira, using a semi-quantitative analysis of immune-stained cells using the following scores: “0”- without any reactivity in parenchymal section, “1” ≤ 10% of positive cells, “2” ≥ 10% of positive cells. It was firm in cell membrane and cytoplasm.

### Statistical Analysis:

The data was collected on specifically designed proforma and analyzed using SPSS version 20.0. Chi Square (χ^2^) Test was used to compare the histological types with expression of immune markers MMP-9 and E-Cadherin. P value ≤ 0.05 was statistically significant.

## RESULTS

Amongst the total of 71 cases, 64 (90%) were freshly diagnosed typical solid / multi-cystic intraosseous ameloblastomas, 7 (10%) cases belonged to uni-cysticin traosseous ameloblastoma. The mean age of patients suffering from ameloblastoma was 34.63±12.16 ranging from 14 to 63 years. Distribution of patients in various age brackets is as follows: 36.6% of patients were between 20 to 30 years of age. 26.6% of patients were between 31 to 40 years of age, whereas, only 3.3% accounted for above 60 years age group. Male patients were 53.5% while the females were 46.5%. Male to female ratio was 1.15:1 (Table-I).

**Table I:** Demographic Characteristics &Ameloblastoma frequency in patients, n = 71.

Ages (mean ± SD)		34.63±12.16	Range (14-63) years
Age brackets, 20-30 years		36.6%	
31-40 years		26.6%	
Above 60 years		3%	
Gender		Male 38 (53.5%)	Female 33 (46.5%)
Ratio		1.15:1	
***Frequency & percent of types of ameloblastoma***			
Acanthomatous		10	14.1%
Unicystic		7	9.9%
Plexiform		17	23.9%
Follicular		37	56.3%
Jaw side	Right	40	56.3%
	Left	31	43.7%
Site	Mandible	59	83.1%
	Maxilla	12	16.9%

The frequency and percentage of cases for different subtypes of ameloblastoma were recorded in this study. Out of 71 patients, Acanthomatous had 10 cases (14.1%), Unicystic had 7 cases (9.9%), Plexiform had 17 cases (23.9%) and Follicular had 37 cases (52.1%). Most ameloblastomas were arising from right side of the jaw involving 40 cases (56.3%) while the left side was involved in 31 cases (43.7%). The mandible was involved in 59 cases (83.1%) while the maxilla was involved in 12 cases (16.9%) (Table-I).

**Fig. 1:**
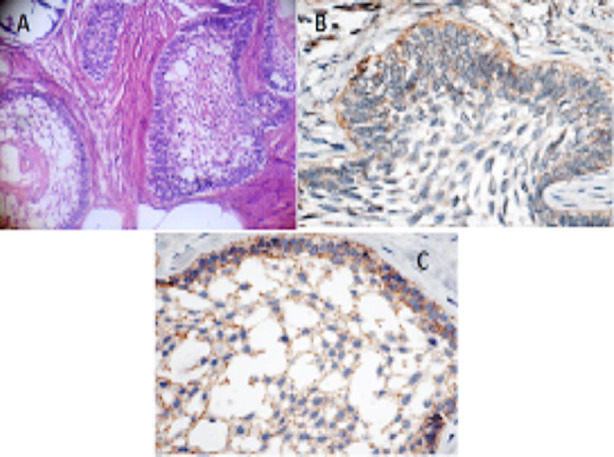
**A:** Follicular HnE, **B:** Follicular MMP-9, **C:** Follicular E-Cad.

**Fig. 2:**
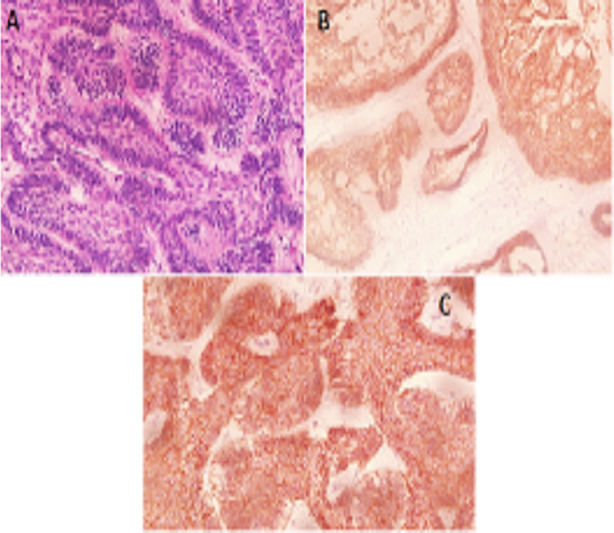
**A:** Plexiform HnE, **B:** Plexiform MMP-9, **C:** Plexiform E-Cad.

**Fig. 3:**
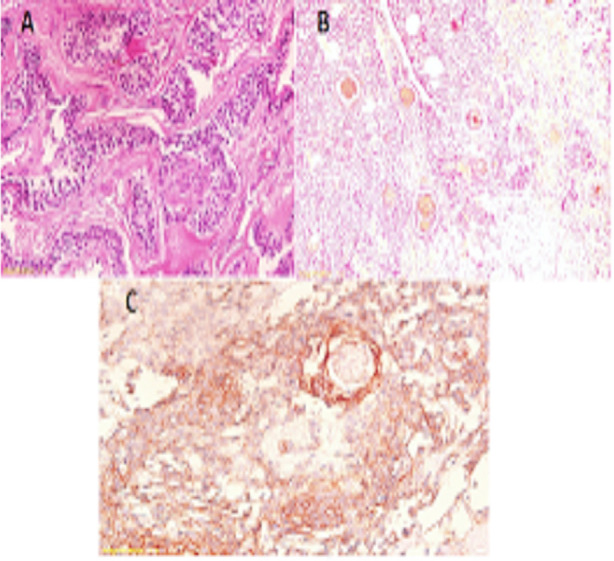
**A:** Acanthomatous HnE, **B:** Acanthomatous MMP-9, **C:** Acanthomatous E-Cad.

The MMP-9 immuno-stain reactivity was evaluated as a percentage. MMP-9 positive cells were counted fields per 400 exaggerations and were expressed. The MMP-9 immuno-reactivity was observed in a diffused pattern both in parenchymal stromal cells. In positive cases, cellular location of MMP-9 was seen in the cytoplasm of parenchymal cells that is stellate like cells (Fig. 1-3).

Amongst 10 cases of Acanthomatous, none was negative. Eight cases showed strong positive results while only two cases showed mild positivity with MMP-9 immuno-stain.

The Unicystic Ameloblastoma was diagnosed in seven cases out of which two case showed strong positivity while 5 cases showed mild positivity.

**Fig.4:**
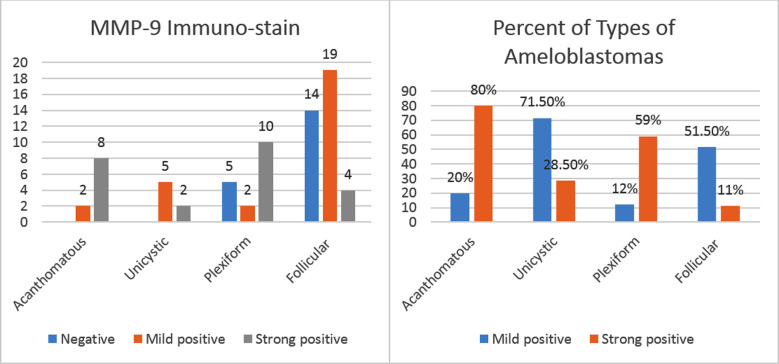
Frequency and percent of types of Ameloblastomas in MMP-9 stain, n = 71.

The Plexiform was diagnosed in 17 cases out of which five cases were negatively stained, two case showed mild positivity and 10 cases appeared strongly positive. The Follicular Ameloblastomas were diagnosed in maximum of 37 cases. Out of these, 14 cases appeared negative, 19 cases exhibited mild positivity and only four cases appeared strongly positive (Fig. 4).

Immuno-Staining of E-Cadherin was controlled in neoplastic epithelial cell of Ameloblastomas and was marked in all examined cases. The most reactivity was observed in stellate reticulum like cells in which the intensity reduced in the peripheral columnar cells especially to the invasive font (Fig. 1-3).

All histological subtypes of Ameloblastomas showed 100% positivity with E-Cadherin immune-stain. Amongst all the positive cases, 44 cases revealed strong positivity while 27 cases were mildly positive (Table-III).

**Table-II:** MMP-9 Expression in Cases of Ameloblastoma, n = 71.

Score of Ameloblastoma	Grading	No. of Cases	Percentage
0 (without any reactivity)	Negative	19	26.7
1 (≤10% of positive cells)	Mild positive	28	39.5
2 (≥ 10% of positive cells)	Strong positive	24	33.8
Total		71	100

p = 0.025.

**Table III:** E-Cadherin Expression in Cases of Ameloblastoma, n = 71.

Score of Ameloblastoma	Grading	No. of Cases	Percentage
0 (without any reactivity)	Negative	0	0
1 (≤10% of positive cells)	Mild positive	27	38
2 (≥ 10% of positive cells)	Strong positive	44	62
Total		71	100

p = 0.375.

**Fig.5:**
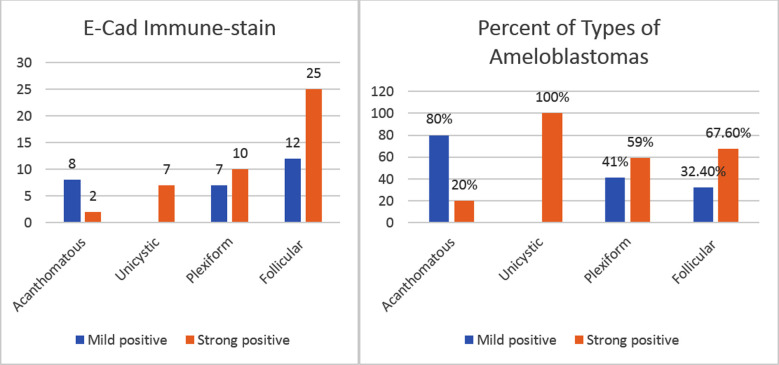
Frequency and percent of types of Ameloblastomas in E-Cad stain, n = 71.

Among the 10 cases of Acanthomatous Ameloblastomas, two cases showed strong positivity while eight cases were found mildly positive. In Unicystic cases, all 7 cases exhibited strong positivity to E-Cadherin immuno-stain. The 17 Plexiform cases were all positive with seven cases revealing mild positivity while 10 cases showed strong positivity. Amongst the 37 Follicular Ameloblastomas, 12 cases were found mildly positive while 25 cases were declared as strongly positive (Fig. 5).

## DISCUSSION

Ameloblastomas are one of the benign epithelial odontogenic tumors arising in the jaw bones. More commonly seen in the mandible than in maxilla, they have the tendency to appear in the posterior part of the mandible. Clinically, Ameloblastomas are divided into many subtypes which are multi-cystic, uni-cystic, peripheral Ameloblastomas amd malignant Ameloblastomas which show invasive behavior.^11^

Our study shares several features common with other published research work and their results. Neville BW et al.^12^ reported a wide range of ameloblastic patients similar to the patients observed in our study that ranged between 14 to 63 years. According to our study, Ameloblastomas occur most commonly in thirty years of life and the mean age of diagnosis is 36.6±12.16 years which is a little later than the study of Chaisuparat R et al.^13^ where signify age was 33.8 years; however, a local study carried out by Khalil E et al.^11^ showed the mean age at diagnosis as 38.01 years. In Pakistan, the late diagnosis may be due to the lack of awareness by the general dental practitioners about the serious consequences and ignorance of patients about their oral health.

Regarding gender, our study revealed slight male predominance with 38 males and 33 females. Male to female ratio was 1.15:1. A study conducted by Chaisuparat R et al.^13^ showed 1.31:1 as the male to female ratio. However, Neville BW et al.^12^ reported equal predilection for males and females. Another study conducted by Hertog and van der Waal showed no sex predilection.^14^

As far as the site is concerned, our study showed mandibular prevalence which is similar to the study conducted by Siar CH et al.^15^ which showed similar results. The results of our study showed that right side is involved more than left side which is same as the outcomes of the study performed by Ladenide AL et al.^16^

Our study also shows similar results to the previous published data in histopathological spectrum. The most common histopathological variant in our study was Follicular Ameloblastoma (52.1%) followed by Plexiform ameloblastoma (23.9%), Acanthomatous Ameloblastoma (14.1%) and Uni-cystic Ameloblastoma (9.9%). A local study conducted by Khalil E et al.,^11^ showed similar frequency amongst the variants of ameloblastoma.

In our study, MMP-9 expression was detected in 52 cases (73.2%) out of a total of 71 cases while 19 cases (26.8%) exhibited negative expression. This is in according to results of Florescu A, et al.,^9^ that found 76.5% ameloblastoma having positive MMP-9 immuno reaction. Henriques AC et al.^17^ also showed 95% positivity in ameloblastoma with MMP-9.

Our research study of MMP-9 analysis proved that cases had score 1 (40%) showing mild positivity, followed by score 2 (33.8%) showing strong positivity. The study outcome of Florescu A et al.^9^ proved majority cases of ameloblastoma with score 1 (47%), followed by the effected cases with score 2 (29.5%).

In current study, a total of 71 cases of ameloblastoma were considered. After applying E-Cadherin, immune-reactivity was observed in all cases ranging from mild positivity (38%) to strong positivity (62%). Very limited studies are available concerning the expression of E-Cadherin on different subtypes of Ameloblastoma. In a study by Kumamto H et al.^18^ both qualitative and quantitative expression of E-Cadherin was high in cells like Central Stellate Reticulum.

### Limitations of the study:

The study was conducted in a single hospital and 71 patients were considered for it. Since Ameloblastoma is not a very common tumor therefore, large number of cases are not available for research purposes.

## CONCLUSION

MMP-9 is considered in the local invasion of ameloblastoma primarily by monitoring extracellular matrix whereas, E-cadherin is concerned in the control of the Ameloblastic local manners by its role in epithelial-mesenchymal transition process. MMP-9 may be used in future as a potential marker to serve as an indicator and utilize in monitoring degree of local aggressiveness of Ameloblastoma. E-Cadherin may not be a very significant marker in cases of ameloblastoma.

### Author’s Contribution:

**FF** provided concept/research design and did data collection.

**ZN and SM** did statistical analysis and manuscript writing.

**FF & ZN** did edit of manuscript and project management.

**BA** did critical revision of the manuscript for important intellectual content.

**ZN & FF** takes the responsibility and is accountable for all aspects of the work in ensuring that questions related to the accuracy or integrity of any part of the work are appropriately investigated and resolved.
